# Inferring Methionine Sulfoxidation and serine Phosphorylation crosstalk from Phylogenetic analyses

**DOI:** 10.1186/s12862-017-1017-9

**Published:** 2017-07-27

**Authors:** Juan Carlos Aledo

**Affiliations:** Departamento de Biología Molecular y Bioquímica, Facultad de Ciencias, Universidad de Málaga, 29071 Málaga, Spain

**Keywords:** Coevolution, Sulfoxide, eIF2α, Oxidative stress, Cytoplasmic stress granules, Post-translational modification

## Abstract

**Background:**

The sulfoxidation of methionine residues within the phosphorylation motif of protein kinase substrates, may provide a mechanism to couple oxidative signals to changes in protein phosphorylation. Herein, we hypothesize that if the residues within a pair of phosphorylatable-sulfoxidable sites are functionally linked, then they might have been coevolving. To test this hypothesis a number of site pairs previously detected on human stress-related proteins has been subjected to analysis using eukaryote ortholog sequences and a phylogenetic approach.

**Results:**

Overall, the results support the conclusion that in the eIF2α protein, serine phosphorylation at position 218 and methionine oxidation at position 222, belong to the same functional network. First, the observed data were much better fitted by Markovian models that assumed coevolution of both sites, with respect to their counterparts assuming independent evolution (*p*-value = 0.003). Second, this conclusion was robust with respect to the methods used to reconstruct the phylogenetic relationship between the 233 eukaryotic species analyzed. Third, the co-distribution of phosphorylatable and sulfoxidable residues at these positions showed multiple origins throughout the evolution of eukaryotes, which further supports the view of an adaptive value for this co-occurrence. Fourth, the possibility that the coevolution of these two sites might be due to structure-driven compensatory mutations was evaluated. The results suggested that factors other than those merely structural were behind the observed coevolution. Finally, the relationship detected between other modifiable site pairs from ataxin-2 (S814-M815), ataxin-2-like (S211-M215) and Pumilio homolog 1 (S124-M125), reinforce the view of a role for phosphorylation-sulfoxidation crosstalk.

**Conclusions:**

For the four stress-related proteins analyzed herein, their respective pairs of PTM sites (phosphorylatable serine and sulfoxidable methionine) were found to be evolving in a correlated fashion, which suggests a relevant role for methionine sulfoxidation and serine phosphorylation crosstalk in the control of protein translation under stress conditions.

**Electronic supplementary material:**

The online version of this article (doi:10.1186/s12862-017-1017-9) contains supplementary material, which is available to authorized users.

## Background

Under stress conditions, cells respond with a global reduction of protein synthesis. Although all the phases of translation are susceptible of being affected, initiation of translation is considered to be the most important regulatory step of the translation cycle [1]. In eukaryotes, translation initiation is a complex and highly regulated process that requires the action of at least a dozen of protein factors. One of these factors, eIF2, is a stable heterotrimeric (subunits α, β and γ) GTPase that binds the Met-RNA_i_^Met^ and delivers it to the small ribosomal subunit. Pairing between the anticodon of the Met-RNA_i_^Met^ and the AUG start codon from the mRNA, triggers hydrolysis of GTP by eIF2 and eIF2⋅GDP is released. Since eIF2⋅GDP cannot bind Met-RNA_i_^Met^, eIF2⋅GDP should be converted to eIF2⋅GTP by the heteropentameric exchange factor eIF2B before it can start a new initiation cycle. In response to stress, phosphorylation of eIF2α on Ser-51 stabilizes the complex eIF2⋅GDP⋅eIF2B and inhibits the GDP-GTP exchange, which prevents the liberation of an active eIF2⋅GTP, thereby reducing initiation of translation [2].

Despite the evidence supporting this description, recent results suggest that the picture is incomplete. In many cases, this mechanism has been presumed to be responsible of the observed inhibition of protein synthesis after stress, simply because eIF2α was found phosphorylated at Ser-51. Indeed, the importance of this mechanism of translation attenuation may have been overestimated as convincingly argued by Knutsen and coworkers [3]. These authors found that when the fission yeast *Schizosaccharomyces pombe* was exposed to hydrogen peroxide, protein synthesis was drastically reduced and eIF2α was phosphorylated on Ser-52 (homologous to Ser-51 in mammalian cells). However, when the mutant eIF2α-S52A was employed in these experiments, the translation was reduced to the same extent as in wild-type cells, despite the inability of this mutant to phosphorylate eIF2α at Ser-52 [3]. Results obtained using other model organisms such as the budding yeast *Saccharomyces cerevisiae* and mammalian cells, also point to the existence of a hitherto unrecognized mechanism contributing to the regulation of translation after stress, independent of the phosphorylation at Ser-51 on eIF2α [3–5].

One possibility, that should not be ruled out, is that residues from eIF2α others than Ser-51 may be post-translationally modified and contribute to the regulation of the translation initiation. In this sense, during a high-throughput study aimed at identifying substrates of kinases related with the cell cycle, eIF2α was found phosphorylated at Ser-218 [6]. However, whether or not this phosphorylation has a functional effect remains yet to be investigated. On the other hand, Met-222 has also been reported to suffer extensive oxidation to methionine sulfoxide (MetO) after treating the cells with H_2_O_2_ [7]. Although oxidation of protein-bound methionine has been traditionally perceived as an inevitable damage derived from aerobic metabolism, it is now emerging as another post-translational modification (PTM) able to regulate protein activity during stress conditions [8]. To this respect, we have recently shown that oxidation of methionine harbored within phosphorylation motifs is a process highly selective among stress-related proteins, including eIF2α and other proteins belonging to stress granules (SGs) [9]. In the current study we have addressed the working hypothesis that both post-translational modifications, sulfoxidation of Met-222 and phosphorylation of Ser-218, may be functionally relevant and interrelated. To this end, we have followed an evolutionary approach.

Molecular coevolution between two positions of a protein occurs when amino acid substitutions at one of these positions affect the rates of substitution at the other position [10]. The forces leading to coevolution derive from functional and/or structural selective pressures acting to maintain specific combinations of residues at the coevolving positions. During the last two decades, much effort has been devoted to investigate molecular coevolution and a plethora of methods aimed at detecting coevolving positions have been described (reviewed in [10, 11]). These can be broadly divided into those methods that attempt to model coevolution in a phylogenetic context and, on the other hand, those methods based on analyzing covariation in multiple sequence alignments. Thus, popular approaches to search for coevolving sites involve substitution correlations [12], mutual information of amino acid frequencies [13] or a global statistical model of the multiple sequence alignment, as is the case of direct coupling analysis [14, 15] and protein sparse inverse covariance [16].

Covariation-based methods, which are simpler and much more popular than those based on phylogenetic grounds, have been shown useful to predict residue contacts [15], and have even proved to be valuable tools for ab initio protein structure predictions. However, as it has recently been brought to our attention by Talavera and coworkers, covariation is a poor measure of molecular coevolution [17]. Therefore, we have followed a phylogenetic approach to study the evolutionary interrelationship between PTM sites. Evidence suggesting a tight relationship between sulfoxidation and phosphorylation among stress-related proteins involved in translation regulation, will be presented and discussed herein.

## Methods

### Phylogenetic trees and multiple sequence alignment data sets

Phylogenetic trees and multiple sequences alignments (MSAs) were initially obtained from eggNOG 4.5, a public resource (http://eggnogdb.embl.de) that provides ortholog groups with integrated functional annotations [18], using the human eIF2α (P05198) as query protein, and the Eukaryota as the target taxon. In this way, 272 sequences belonging to 233 species were retrieved. When multiple paralogs from one species were available, only the one with the smallest editing distance to the human homolog was included. Similarly, the original tree was pruned to reflect the phylogenetic relationship between the 233 ortholog proteins. In addition to the pre-computed tree from eggNOG, the phylogenetic relationship between these 233 sequences was also reconstructed using the neighbor-joining (NJ) [19]. Trees based on maximum parsimony (MP) employing the Fitch algorithm and the nearest neighbor interchange rearrangement strategy, and trees based on maximum likelihood analysis using a general time-reversible model with four discrete gamma rate categories, were computed with the assistance of two R packages: *phangorn* 2.0.4 [20] and *ape* 3.5 [21]. All these trees, as well as the MSA and raw data related to eIF2α, can be downloaded from https://github.com/jcaledo/PTM_sites_coevolution. Trees and MSAs for other SG related proteins such as ataxin-2 (Q99700), ataxin-2-like (Q8WWM7) and Pumilio homolog 1 (Q14671) were obtained from eggNOG 4.5. When the MSAs were not intended to reconstruct phylogenetic relationships, but to compute the state (the amino acid found) at different positions in the ortholog proteins, the MSA was further modified to remove those columns corresponding with gaps in the human protein used as reference.

### A four-state continuous-time Markov chain model of evolution

**Fig. 1:**
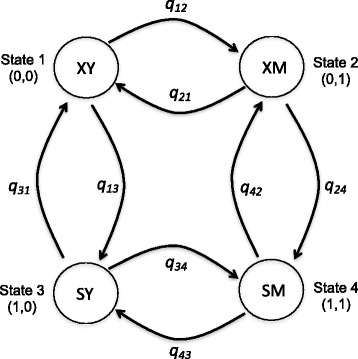
Diagram of the Markovian model of evolution for a pair of PTM sites. For two binary traits, four combined states are possible. Each state is defined by an ordered pair where the first entry is either 0 or 1 depending on the absence or presence, respectively, of a phophorylatable residue at the considered position (trait X). The second entry of the pair again will be 0 or 1 depending now on the absence or presence of a methionyl residue at the analyzed position (trait Y). The allowed transitions between states are indicated by the *arrows* and their associated rate parameters, q_ij_

To model the evolution of the residues found at positions 218 and 222, we used the theoretical framework of continuous time Markov processes. Since proteins are built up from a pool of twenty proteinogenic amino acids, the state space of these markovian models should, supposedly, be composed by 400 elements. However, most sites in ortholog proteins generally exist in a limited number of residue states. Furthermore, because our interest was focused on detecting coevolving PTM sites, the cardinality of the state space can be drastically reduced. Thus, for the character residue at position 218 (random variable X) two states are possible. When the residue found is a phosphorylatable one we set X = 1, and X = 0 otherwise. Similarly, for the character residue at position 222 (random variable Y) the accessible states are also two: a sulfoxidable methionine is found at that position (Y = 1) or any other non sulfoxidable amino acid is observed at that position (Y = 0). Four combinations of states are possible when these two binary variables are simultaneously considered. Each of these four states can either stay the same over the length of a branch of the phylogeny, or change to one of the three other states. Fig. 1 links the four combinations of states by arrows with parameters that describe the evolutionary rates of transitions between two states of one character, holding constant the state of the other. This Markov process is defined by the instantaneous rate matrix:

1 $$ \boldsymbol{Q}=\left(\begin{array}{cc}\begin{array}{cc}-\sum {q}_{1j}& {q}_{12}\\ {}{q}_{21}& -\sum {q}_{2j}\end{array}& \begin{array}{cc}{q}_{13}\kern2.75em & 0\\ {}0\kern3.25em & {q}_{24}\end{array}\\ {}\begin{array}{cc}{q}_{31}\kern2.5em & 0\\ {}0\kern2.75em & {q}_{42}\end{array}& \begin{array}{cc}-\sum {q}_{3j}& {q}_{34}\\ {}{q}_{43}& -\sum {q}_{4j}\end{array}\end{array}\right) $$

The values of the rate parameters describing instantaneous double changes, such as (0,0) → (1,1), (0,1) → (1,0), (1,0) → (0,1) and (1,1) → (0,0) are set to zero because the probability of both traits changing in the same instant (*dt*) is negligibly small and can be ignored. The model, however, allows both traits to change over a longer time period, *t*, but they must have transitioned through intermediate states. This approach is particularly suitable to detect evolutionary relationship between two traits, in which the state of one trait affects the probability of a change in the other [22].

The constraints that each row sums to zero, and that dual transitions have a probability of zero over time *dt*, means that the rate matrix, ***Q***, defining the Markov process, is fully specified by eight parameters. However, if the two traits have evolved independently of one another, then the rate of change between the two states of one character will not depend on the background state of the other. For instance, if the rate of gaining an oxidable methionine at position 222 does not depend on the amino acid found at position 218, then *q*_*12*_ *= q*_*34*_. More generally, the model of independent evolution can be defined by setting *q*_*12*_ *= q*_*34*_ *, q*_*13*_ *= q*_*24*_ *, q*_*21*_ *= q*_*43*_ and *q*_*31*_ *= q*_*42*_ *.* Thus, the model of independent evolution uses a maximum of four parameters, while the model of dependent evolution does not place any restrictions on the parameters, allowing some kinds of transitions to depend on the background state of the other trait. When that happens, pairs of states will tend to be associated with each other in the species data more often than expected by chance, and the dependent model will provide a better description of the data.

### Likelihood function optimization and likelihood ratio test

Given the assumption of either independence (***Q***_***I***_) or dependence (***Q***_***D***_) between X and Y, the corresponding substitution probability matrices can be calculated using **P**_**I**_(t) = e^(***Q***^_***I***_^*t*)^ and **P**_**D**_(t) = e^(***Q***^_***D***_^*t*)^, respectively. Eigenvalues and eigenvectors necessary for determining these expressions were calculated using standard numerical methods. To find the likelihood of our data given a chosen model, we have to consider all possible assignments of character states at the interior nodes. The likelihood of each of these single realizations is given by the product over all of the branches of the tree of the appropriate probabilities, as derived from the previously computed substitution probability matrices using the appropriate branch lengths. The likelihood of the data will be the sum over all possible realizations, which will be expressed as a function of the model parameters. In the Appendix A, from Additional file 1, we have considered a simple hypothetical phylogeny and data set to illustrate these calculations. The maximum likelihood estimates of parameters are the parameter values that maximize the likelihood function, and were found numerically using iterative optimization algorithms implemented in R [23]. Once the likelihoods for the independence *L(I)* and dependence model *L(D)* were computed, the value of the likelihood ratio test statistic was obtained according to the following equation:

2 $$ LRT=-2\mathit{\ln}\frac{L(I)}{L(D)} $$

Although we routinely use the R function *ace* from the package *ape* [21] to fit the so-called M*k* models (in our case M4 models) other functions such as *fitDiscrete* (from the package *GEIGER*) [23] and *fitMk* from the package *phytools* [24] were also used yielding similar results. Different assumptions regarding the stationary frequencies of each character state, only involved slight differences in parameter estimates and likelihoods. Thus, regardless of the frequency vector used (‘equal frequencies’, ‘estimated frequencies from the stationary distribution of ***Q***’ or ‘observed frequencies’) the conclusion was always the same: the dependent models of evolution explained much better the data than the independent models (LRT = 15.9, 15.4 and 15.6, respectively).

### Pairwise comparison analyses

Using the multiple protein sequence alignment of eIF2α, pairs of species contrasting in both binary characters (absence/presence of phosphorylatable residue at 218 and absence/presence of sulfoxidable residues at 222) were searched and analyzed as described by Maddison [25] and implemented in Mesquite 3.10.

### Stochastic character mapping

We used stochastic character mapping [26] to infer 10 possible evolutionary histories of residues at positions 218 and 222 on the phylogenetic tree of eukaryotic IF2α protein. To this purpose we employed the function *make.simmap* in the *phytools* package (v. 0.5–38) for R [24]. For the parametrization of *make.simmap*, we used a model of unequal rates. To check the robustness of the obtained results, we tested two methods, Q = “empirical”, which first fits a continuous-time Markov model for the evolution of our combined characters, and then simulates stochastic character histories using that model and the data (the tip states on the tree). Alternatively, Q = “mcmc”, first samples Q 10 times from the posterior probability distribution of Q using Markov chain Monte Carlo, and then it simulates 10 stochastic maps conditioned on each sample value of Q. Apart from slightly higher variances in the number of transitions between states through the simulated histories, both methods provided similar results.

### Protein stability of double-mutants

The thermodynamic stability changes (∆∆G) of single and double-mutants at different positions of eIF2α were computed using the protein design tool FoldX version 4.0 [27]. FoldX uses a full atomic description of the protein structure to provide a quantitative estimation of the importance of the interactions contributing to the stability of the protein. For this purpose, the different energy terms taken into account, which have been described in detail somewhere else [28], have been weighted using empirical data obtained from protein engineering experiments. The 3D structure of eIF2α (1Q8K) was subjected to an optimization procedure using the RepairPDB command from FoldX. Afterwards, 400 double-mutant models were built for each pair of positions. For instance, for the study of the pair S218X-M222Y, 400 models were built and analyzed (with X and Y belonging to the set of twenty proteinogenic amino acids).

## Results

### Residues at positions 218 and 222 from eIF2α have been coevolving

Two positions of a protein are said to be coevolving if they mutually influence their evolutionary rates. As it has already been pointed in the Introduction, covariation is a poor measure of molecular coevolution [17]. Therefore, we rather resorted to a maximum likelihood methodology for detecting correlated evolution on phylogenies. The method, which is an adaptation of that originally proposed by Mark Pagel in a seminal work published in 1994 [22], relies on a model based on Markov chain in continuous time and the optimization of the associated likelihood function to obtain the model’s parameters that best fit the data. Our data set encompasses a phylogenetic tree of the eIF2α protein from 233 eukaryotic species, including branch lengths, as well as information about the residues found at positions 218 (character or variable X) and 222 (character or variable Y) in these ortholog proteins (Additional file 1: Figure S1).

**Fig. 2:**
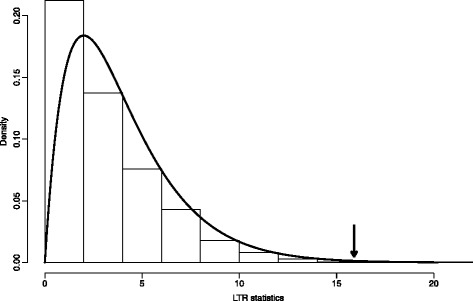
Comparing models of evolution for the S218-M222 PTM site pair on eIF2α. The compared models were one assuming independent evolution of both PTM sites (null model), and an other assuming coevolution (alternative model). The likelihood value under a given model measures the fit of that model to data. Hence, the two models can be compared by comparing their respective likelihood values. The computed LRT statistic value for the independent versus dependent evolution was 15.9 (*arrow*). Since in our case both models were nested (see Methods), the probability distribution of the LRT statistic, assuming that the null model is true, can be approximated by a χ^2^ distribution with four degree of freedom (*black* *continuous curve*). In this way, the null model (independent evolution of both sites) could be rejected with a *p*-value of 0.003. The same conclusion was reached when the distribution of the LRT statistic was derived by Monte Carlo simulation (histogram bars)

To test for correlated evolution between variables X and Y, we used a likelihood ratio test (LRT) to compare the performance of two models, one assuming independent evolution of the characters and one assuming coevolution (see Methods). The likelihood ratio test significantly supported the dependent model (LRT = 15.9, df = 4, *p* = 0.003). Although the χ^2^ distribution is the asymptotic distribution for the LRT statistic when the compared models are nested, it may not always apply directly. Whether the LRT statistic is distributed as a χ^2^ may depend upon the values of the rate parameters and the amount of data [22], as well as the tree structure and the equilibrium state frequencies [29]. Therefore, to conclude with confidence that the coevolutionary model is better model for our data, we turned to the Monte Carlo parametric bootstrapping technique. Briefly, we started by finding the maximum likelihood estimates (MLE) of the four parameters of the model of independent evolution that best fitted to our data. These MLE parameters were then used to evolve the two characters along the established phylogeny in 1000 simulations. Using the data from these simulations, the dependent and independent models were fitted and their likelihoods computed. The likelihood ratios, of the two models for each simulation, were used to form the null distribution that is shown in Fig. 2 as a bar histogram plot. As it can be observed, the empirical Monte Carlo distribution of the statistics LRT matched quite closely the chi-squared distribution with four degree of freedom. Therefore, the model of independent evolution can be rejected with high confidence in favor of the coevolution model.

### The evolutionary codependence between positions 218 and 222 is linked to PTM

The existence of correlated evolution between the sites 218 and 222 has been examined above by linking to each position a biological character. Concretely, the presence/absence of phosphorylatable residues at 218 and the presence/absence of a sulfoxidable amino acid at 222. Thus, the concluded codependence may be related to the PTMs studied. However, residue-residue interactions unrelated to the phosphorylation-sulfoxidation interplay may lead to the observed coevolutionary signal when collapsing residues in a reduced dictionary. To investigate this possibility, we have explored the codependence between these sites using different sets of residues to define the model states. For instance, one of the different alternative that were assessed was: X = 1 if at position 218 is found a residue sensitive to deamidation (asparagine or glutamine) and Y = 1 if the non-polar valine is present at position 222. In this way, the evolutionary codependence of these traits, X and Y, was studied for 16 alternative definitions of states. As it can be observed in Table 1, none of these alternative definitions led to a significant coevolutionary signal. This lack of codependence, in these other reduced dictionaries, support a link between the coevolution at these positions and the PTMs undergone for the residues present at these sites.

**Table 1:** Sets of residues unrelated to the crosstalk between phosphorylation and sulfoxidation do not show coevolutionary signal

Group	Set at 218	Set at 222	LRT	*p*-value
Positive	{S,T}	{M}	15.9	0.003
Control-1	{S,T}	{V}	2.4	0.668
Control-1	{S,T}	{C}	3.1	0.542
Control-1	{S,T}	{N}	4.6	0.336
Control-1	{S,T}	{L}	0.0	1.000
Control-2	{N,Q}	{M}	3.7	0.449
Control-2	{G,A}	{M}	0.0	1.000
Control-2	{D,E}	{M}	0.0	1.000
Control-2	{H,K}	{M}	0.0	1.000
Control-3	{Q,N}	{V}	0.2	0.993
Control-3	{G,A}	{V}	6.2	0.181
Control-3	{D,E}	{V}	0.0	1.000
Control-3	{H,K}	{V}	3.5	0.475
Control-4	{Q,P}	{G}	0.0	1.000
Control-4	{W,V}	{A}	0.0	1.000
Control-4	{L,T}	{I}	0.0	1.000
Control-4	{I,Y}	{H}	0.0	1.000

The existence of correlated evolution between the sites 218 and 222 was examined using different sets of residues to define the model states. Thus, beside the sets formed by phosphorylatable residues at 218 and sulfoxidable methionine at 222, used as positive reference, four other types of set combinations were analyzed. In the first type (Control-1, rows 2–5), the residues providing X = 1 are still serine and threonine, but the residue making Y = 1 is now different to methionine (as indicated in the table). Val, Cys, Asn and Leu have been selected because of their frequencies (from higher to lower) at position 222 in the eukaryotic species examined. The second group of control analyses (Control-2, rows 6–9) always included methionine at position 222 as the residue providing Y = 1, while the set leading to X = 1 was now formed by a pair of non-phosphoaceptor amino acids of similar physicochemical properties. The third group (Control-3, rows 10–13) was formed by sets of residues that were neither phosphorylatable nor sulfoxidable. Finally, the fourth group (Control-4, last four rows), was established by randomly taking the sets, among the twenty proteinogenic amino acids. The LRTs obtained, and their respective *p*-values, are shown

### Robustness of the coevolution model with respect to the phylogeny

A fundamental assumption of the maximum likelihood approach is a correct topology of the used tree, as well as an accurate estimation of their branch lengths [22]. To check the robustness of our conclusion regarding the evolutionary relationship between Ser-218 and Met-222, we carried out the coevolutionary analysis described above, but employing trees that were reconstructed using different methods. The results of such analyses are summarized in Table 2. Although the trees obtained using methods based on genetic distances, maximum parsimony and maximum likelihood were slightly different between them, in all the cases we concluded that the model of coevolution between both PTM sites fit the observed data significantly better than the model of independent evolution, regardless of the method used to reconstruct the phylogeny.

**Table 2:** Influence of the phylogeny on the likelihood ratio test between the models of dependent and independent changes

Tree	Ln(L)	Fitch score	Topology difference	Branch score	LRT	*p*-Value
eggNOG	−43,009	7499	0	0	15.9	0.0030
NJ	−43,677	7596	198	1.664	20.8	0.0003
MP	−60,127	7472	28	426.745	14.1	0.0070
ML	−37,544	7511	111	1.118	17.1	0.0020

Besides the pre-computed tree from eggNOG, trees reconstructed using the methods of neighbor-joining (NJ), maximum parsimony (MP) and maximum likelihood (ML), were used to test the hypothesis of correlated evolution between Ser-218 and Met-222. In addition to the LRT and its related *p*-value, the table shows the natural logarithm of the likelihood, Ln(L), and the Fitch score for each tree. The distance between each tree and that from eggNOG was assessed using either the metric proposed by [53] (topology difference) or the branch score proposed by [54]. The former, is defined as twice the number of internal branches that differ in their splits, while the latter is defined as the sum of squares of the differences between each branch’s length in both trees

### Reliability of the maximum likelihood approach to detect functional PTMs

Residues that are close in the three-dimensional structure of the protein are expected to mutually influence their evolution more often than residues that are far away from each other, merely due to structural reasons [29]. Since both PTM sites (Ser-218 and Met-222) are four residues away from each other, we wanted to explore whether causes other than structural may underline the observed coevolution between them. To this end, we carried out the following control analyses.

**Fig. 3:**
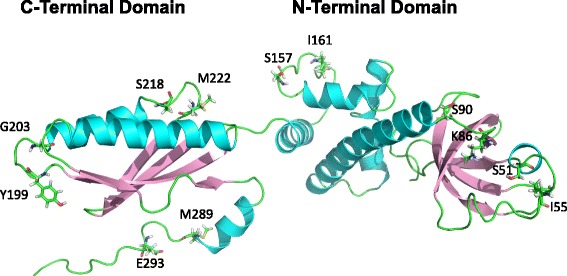
Spatial distribution of structurally comparable residue pairs from eIF2α. Ribbon cartoon of the three-dimensional structure of the α subunit of human eIF2 (PDB 1Q8K). The modifiable residues (either Ser, Thr, Tyr or Met) located outside helices and strands are displayed using stick representation, as well as those amino acids found four positions away from them

Ser-218 and Met-222 are located in a loop between helix α6 and strand β7 from the C-terminal domain of eIF2α. Thus, we selected all the pair sites found outside helices and strands that included a modifiable residue (either Ser, Thr, Tyr or Met) together with the amino acid found four positions downstream from it, but still remaining within the same loop (Fig. 3). In this way, five control site pairs were selected, including a positive control site pair such as that formed by Ser-51 and the hydrophobic residue Ile-55, which is expected to show a high degree of coevolution due to the well known functional relevance of these sites [30]. On the other hand, the remaining four site pairs are referred to as negative control because they include non-regulatory sites [31]. That is, residues that may be modified only as consequence of off-target interactions and therefore are thought to be of little, if any, functional significance [32]. All these control site pairs were subjected to analysis using the same maximum likelihood methodology used to analyze the pair Ser-218/Met-222 under study. The results of such coevolutionary analyses are summarized in Table 3. As it can be observed, data related to non-functional site pairs are best explained by assuming independent evolution, in spite of the proximity between the sites. In contrast, the functional pair Ser-51/Ile-55, used as positive control, yielded a high LRT indicative of the good performance of the current procedure to detect functionally important PTMs.

**Table 3:** Sites from non-regulatory pairs evolve independently despite their spatial proximity

Group	Sites	Regulatory	Loop	Distance	∆∆G	LTR	*p*-value
A	S51-I55	Yes	β3/β4	6.2	−0.6 ± 1.2	27.7	1.5 10^−5^
A	S90-K86	No	β5/α1	9.2	2.1 ± 1.3	5.4	0.242
A	S157-I161	No	α4/α5	9.7	0.0 ± 0.8	3.9	0.420
A	Y199-G203	No	β6/α6	8.8	9.7 ± 4.8	0.0	1.000
A	S218-M222	Yes?	α6/β7	6.3	2.2 ± 2.0	15.9	0.003
A	M289-E293	No	CT	9.5	−0.3 ± 0.8	5.9	0.207
B	C217-M222	No	α6/β7	9.9	1.2 ± 2.1	2.1	0.712
B	S218-M222	Yes?	α6/β7	6.3	2.2 ± 2.0	15.9	0.003
B	T219-M222	No	α6/β7	4.9	0.6 ± 1.6	0.0	1.000
B	E220-M222	No	α6/β7	6.7	1.4 ± 1.6	6.5	0.164
B	N221-M222	No	α6/β7	5.3	1.5 ± 1.9	6.6	0.158
B	P223-M222	No	α6/β7	5.3	2.1 ± 1.5	10.5	0.033
C	S218-C217	No	α6/β7	4.4	1.6 ± 1.9	8.5	0.075
C	S218-T219	?	α6/β7	4.4	0.5 ± 1.4	17.9	0.001
C	S218-E220	No	α6/β7	8.4	1.7 ± 1.3	12.9	0.012
C	S218-N221	No	α6/β7	9.7	2.0 ± 1.8	8.1	0.088
C	S218-M222	Yes?	α6/β7	6.3	2.2 ± 2.0	15.9	0.003
C	S218-P223	No	α6/β7	7.7	2.6 ± 1.4	10.5	0.033

The LRTs between the models of correlated and uncorrelated evolution were computed for the indicated site pair, as well as their associated *p*-values. The distances, in ångströms, between residues are also given. The pairs shown in the upper part of the table (Group A) are those whose members are four residues away from each other and they are outside helices and strands. Three of these tested site pairs were found within the N-terminal domain (NTD), while the other three were located in the C-terminal domain (CTD) of the eIF2α protein. In the middle (Group B) and lower (Group C) parts of the table, the relationships between M222 and its neighbors and S218 and its neighbors, respectively, are analyzed. For each pair of sites, the thermodynamic stability change (ΔΔG) for the 400 possible double-mutants was computed and the mean ± standard deviation is shown in kcal/mol

**Fig. 4:**
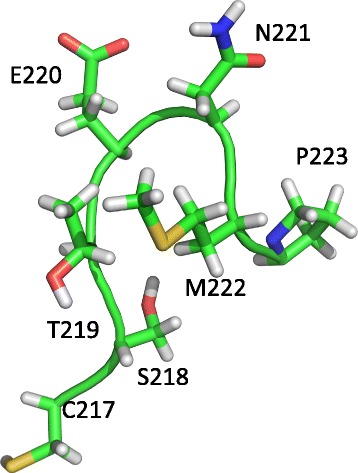
Structure of the α6/β7 loop from eIF2α. The spatial disposition of the residues forming α6/β7 is shown using stick representation

To further explore the possibility that the three-dimensional distance between these pairs of residues may influence the observed coevolutionary signal, we extended the set of control pairs to include all the pairs formed by a target residue (either Ser-218 or Met-222) and a second residue in the vicinity within the α6/β7 loop (Fig. 4). Using all these pairs, we failed to detect any relationship between the distance (in ångströms) separating the pair members and their evolutionary codependence, as estimated by their LRT statistic values (Table 3 and Additional file 1: Figure S2). In addition, we also addressed whether the structural importance of the pair of sites might be a key determinant of their evolutionary codependence. To this end, for each pair of sites the mean thermodynamic stability change (ΔΔG) for the 400 possible double-mutants was computed and plotted against its LRT. Again we failed to observe a relationship between these variables (Additional file 1: Figure S3).

### Vigilance against over-interpretation

**Fig. 5:**
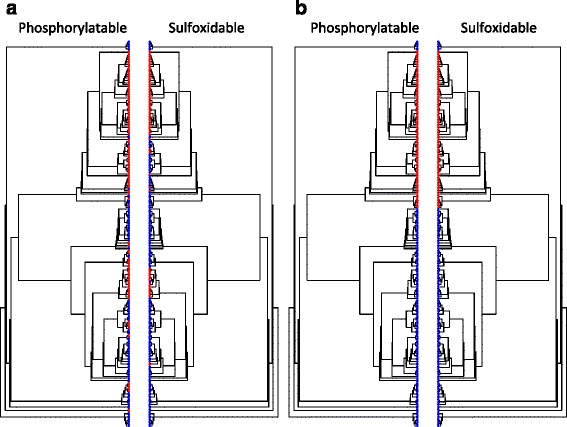
Co-distribution of phosphorylatable and sulfoxidable residues through eukaryote evolution. **a**. The same phylogeny of eIF2α is mirrored to show the pattern of co-distribution of phosphorylatable and sulfoxidable residues. In the rightwards tree, the presence/absence of a phosphorylatable residue at position 218 of eIF2α in that eukaryotic species is indicated by a *red/blue dot*, respectively. Similarly, the presence/absence of a sulfoxidable residue at position 222 is indicated by a *red/blue dot* in the leftwards tree. **b**. Hypothetical co-distribution of both characters, made up to illustrate Darwin’s scenario: when the co-distribution of specific states of two characters is the result of sharing a pair of synapomorphic characters, each emerged independently and then maintained each by its own motives in the descendants

In a recent work Maddison and Fitzjohn warn against the risks of over-interpreting the results of phylogenetic correlation tests based on Pagel’s method. Although these well-respected methods are commonly used in many biological fields, they do not eliminate pseudo-replications derived from a single evolutionary event [33]. They argue that, in some circumstances, the co-distribution between two characters along the phylogeny may be the result of sharing a pair of synapomorphic characters, each emerged by its own independent causes in a common ancestor, and then maintained each by its own causes in the descendants. Such a situation is what these authors call Darwin’s scenario. Therefore, if we want to support the conclusion that serine phosphorylation and methionine oxidation form part of the same functional network, Darwin’s scenario should be ruled out. In Fig. 5a the same phylogeny of eIF2α is mirrored to show the state of character X (absence/presence of phosphorylatable residue) at left, and Y (absence/presence of methionine) at right. On the other hand, Fig. 5b shows a hypothetical co-distribution of both characters made up to illustrate Darwin’s scenario. As it can be deduced from this figure, the co-distribution of phosphorylatable and sulfoxidable residues throughout eukaryotes does not seem to fit in Darwin’s scenario.

**Fig. 6:**
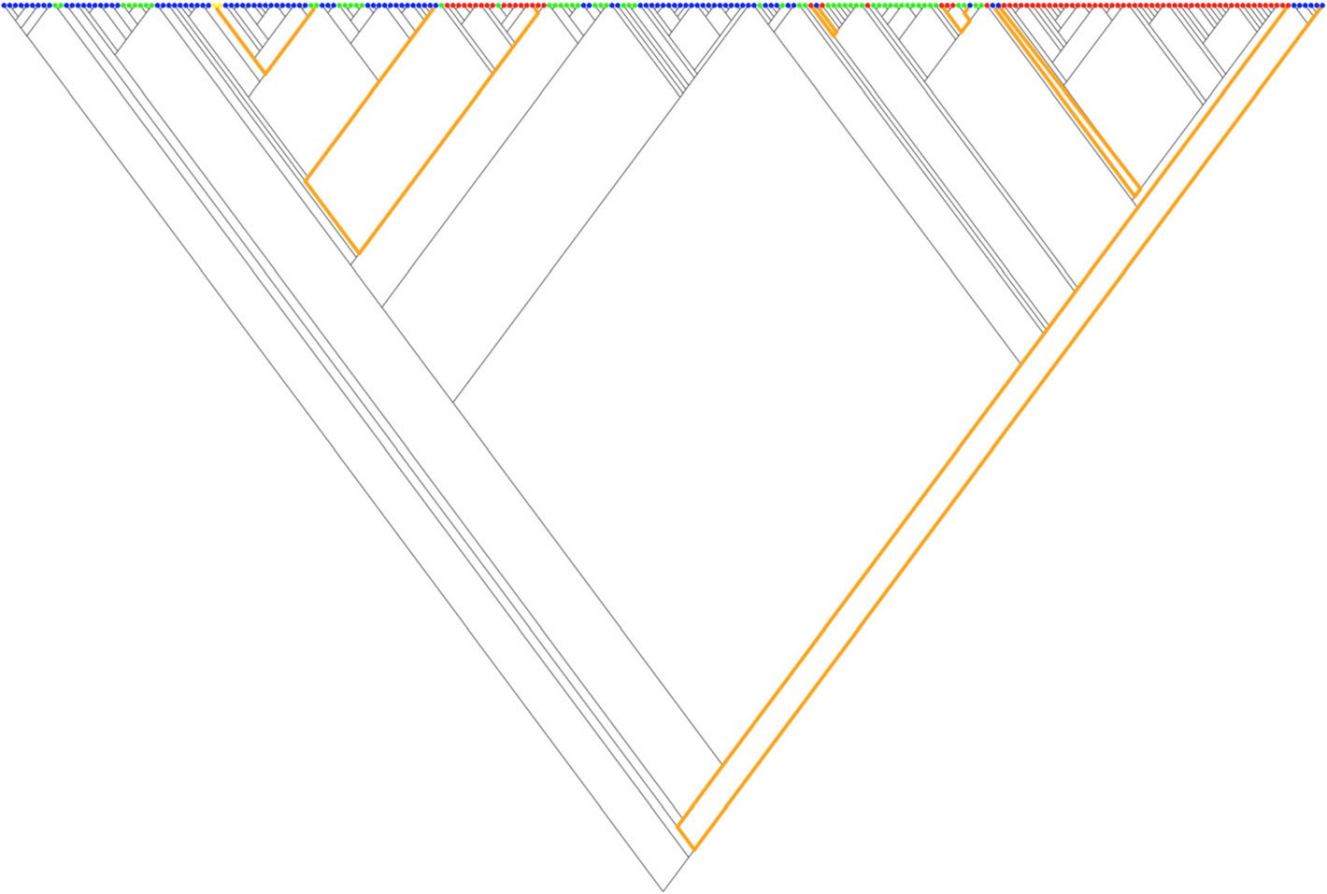
Separate evolutionary origins of the co-occurrence of modifiable residues at positions 218 and 222 of eIF2α. The combined character states were encoded with colors as follows. Blue (state 1: (0,0)); yellow (state 2: (0,1)); green (state 3: (1,0)); red (state 4: (1,1)). Those pairs of species that differed in both characters and were phylogenetically separate (the path between them along the branches of the tree do not touch the path of any other pair) are shown connected by a thick orange line. Since we are examining pairs contrasting in both characters, there are two types of possible pairs: a pair is considered to be positive when the presence of a phosphorylatable residue in a member of the pair is accompanied by a methionine in the same member of the pair {red, blue}, otherwise the pair is referred to as negative {yellow, green}. In addition, to show that the co-occurrence of both PTMs has multiple and independent evolutionary origins, it can also be noted that the number of positive pairs is greater than the number of negative pairs

In an attempt to numerically support this graphic result, we resorted to pairwise comparisons of terminal taxa to test for character correlation [25]. Only pairs of taxa that differed in both characters and were phylogenetically separate were considered. A pair is said to be phylogenetically separate if the path between the members of the pair, along the branches of the tree, do not touch the path of any other pair (Fig. 6). Since we are examining pairs contrasting in both characters, there are two types of possible pairs: a pair is considered to be positive when the presence of a phosphorylatable residue in a member of the pair is accompanied by a methionine in the same member of the pair ({(1,1), (0,0)}), otherwise the pair is referred to as negative ({(1,0), (0,1)}. The idea underlying this analysis is simple. When pairs of species contrasting in the state of a particular character are examined, the member of a pair with a particular state might be more likely than the other member to exhibit a particular state in the second character. In other words, the null hypothesis states that the number of positive pairs is equal to the number of negative pairs. When we subjected our data to such an analysis, we found that many different maximal pairings could be defined (a maximal pairing is a set of phylogenetically separate pairs of terminal taxa that contains the most pairs possible for the given tree). In all the cases, these pairings contained five positive pairs and one negative pair (Fig. 6). Pairwise comparison analysis can avoid the pitfalls of being influenced by a single origin of a character state by choosing pairs of taxa that contrast in the states of both variables. However, the method uses only a small subset of taxa, discarding much of the data, and so it has a very low power to detect correlations [33]. That seems to be the case with our data. Despite that the number of positive pairs overtook the number of negative ones (five to one), the difference did not reach statistical significance due to the low number of total phylogenetically separate pairs (only six). Nevertheless, the fact that among contrasting pairs the probability of being positive is five times greater than that of being negative allows, at least, to rule out a single origin of the phosphorylatable-sulfoxidable co-distribution among eukaryotes.

### Stochastic character mapping also supports coevolution

To tackle the potential problem of non-independence in phylogenies using a different approach, we took advantage of the idea underlying Ridley’s method, which was specifically tailored to get around the problem of pseudo-replications [34]. In the original description of the method, the character states of internal nodes are reconstructed using parsimony. Once the internal nodes have been assigned, one works through the phylogeny keeping a tally of the number of transitions in the tree. More concretely, the method scores transitions only in those branches for which the beginning and end-states differ. That is, branches along which no change occurs are not included. By not including such branches, the method avoids counting species or internal nodes that share character states with an immediate common ancestor, and which thereby cannot be considered independent data points. Herein, instead of reconstructing the states of both characters at internal nodes using parsimony, which have a number of serious limitations [26], we rather carried out stochastic character mapping using a Markov chain Monte Carlo approach to sample character histories from their posterior probability distribution. In this way, after simulating 10 stochastic character histories, a contingency table showing the number of branches along which transitions occurred that ended in each of the four possible states, {(0,0), (0,1), (1,0), (1,1)}, could be computed (173, 22, 285 and 68, respectively). Making use of such contingency table, the null hypothesis that changes at position 218 (character X) and 222 (character Y) were independent was rejected (*p*-values = 0.021 and 0.016, for Yates’ chi-squared and Fisher’s exact tests, respectively).

### Phosphorylation-sulfoxidation relationship in other stress-related proteins

For most protein kinases, the selection of target substrates is strongly influenced by the amino acid sequence surrounding the phospho-acceptor site [35]. The amino acids within these environments that either promote or compromise the phosphorylation are referred to as specificity determinants. In a recent work, we investigated those phosphorylation motifs where methionine may play a role as specificity determinant, finding that the reversible oxidation of methionines located at one (P + 1) or four (P + 4) positions carboxyl-terminal to the phosphosite was a process highly selective among stress-related proteins, which may couple oxidative signals with changes in protein phosphorylation [9]. In this way, besides eIF2α other three proteins constituents of SGs, such as ataxin-2, ataxin-2-like and pumilio homolog 1, were identified as potential targets for crosstalk between sulfoxidation and phosphorylation [9]. In humans, each of these proteins contains a serine that has been proved to be phosphorylatable [36] and that is accompanied by a methionine either at P + 1 or P + 4. In addition, these methionine residues are known to be oxidized in vivo after an oxidative stimulus [7]. Herein, the coevolution of these PTM site pairs (phosphorylatable serine and sulfoxidable methionine) was evaluated by the markovian-likelihood method described above for eIF2α. Table 4 summarizes the results of such analyses. As it can be observed, the evolution of the PTM site pairs was significantly better explained, in all the cases, by the dependent evolution model when compared to the model that assumed independent evolution.

**Table 4:** Testing for correlated evolution between PTM sites from SG

Protein	Sites	*l(I)*	*l(D)*	LRT	*p*-Value	N
eIF2α	S218-M222	−108.2	−100.2	15.9	0.003	233
Ataxin-2	S814-M815	−95.3	−89.8	10.9	0.027	215
Ataxin-2-like	S211-M215	−135.0	−120.8	28.3	10^−5^	215
Pumilio homolog 1	S124-M125	−38.9	−23.3	31.2	2.8 10^−6^	51

The column ‘Sites’ gives the residue (S: serine and M: methionine) found at the indicated position in the human ortholog sequence. LRT stands for the likelihood ratio test statistics and N for the number of species included in the analyses. *l(I)*: natural logarithm of the likelihood value for the model of independent evolution. *l(D)*: natural logarithm of the likelihood value for the model of dependent evolution

## Discussion

Many of the cellular responses triggered by oxidative stress are known to be mediated by signaling cascades involving protein phosphorylation [37, 38]. Despite the enormous research effort that has been devoted to the study of protein phosphorylation, the molecular mechanisms coupling oxidative signals to changes in phosphorylation remain poorly understood. A direct way through which oxidants may be sensed and transduced into biological responses involves reversible oxidation of protein-bound methionine to MetO [8]. Like phosphorylation, methionine oxidation is a reversible covalent PTM that can impact protein function in different ways. Thus, it has been shown that sulfoxidation of specific methionine residues can determine the subcellular distribution and activity of the target protein [39–41]. In yet another parallelism with phosphorylation, methionine oxidation can lead to either down-regulation [42, 43] or up-regulation [44, 45] of protein activity. In addition, both methionine oxidation and methionine sulfoxide reduction are reactions that can be enzyme-catalyzed [46, 47]. In this context, it has been proposed that oxidation of methionine, that converts the side chain of this amino acid from hydrophobic to hydrophilic [48], may provide the basis for regulating the specificity of protein kinase-substrate interactions [49, 50]. In a previous study, we addressed the potential for crosstalk between sulfoxidation and phosphorylation at the proteome scale, reaching the conclusion that the interplay between serine phosphorylation and methionine oxidation was most prevalent among proteins involved in the control of translation during stress response. However, no study, hitherto, has developed an evolutionary approach to address the potential crosstalk between these two PTM types. To this respect, the original rationale for the current work was that if two PTM sites are functionally related and they are modified in a coordinated fashion, then they might have been coevolving over time.

Among the wide range of computational methods that have been proposed to detect coevolving residues (reviewed in [11, 51]), several exist that attempt to model coevolution in a phylogenetic context [29, 52]. However, far more popular methods are those that search for covariation between sites in a tree-independent manner. Although the need to account for the phylogenetic relationship is a well-acknowledged fact among evolutionary biologists, it has been poorly addressed, or simply ignored, by those authors more biased toward functional and/or structural biology. However, because molecular sequences share common ancestries and are therefore not independent from each other, the underlying evolutionary history of sequences should be taken into account if we pretend to properly extract the coevolutionary signal out of the noise. Therefore, even though less popular, conceptually more complex and computationally expensive, we resorted to a phylogenetic method to assess whether the two eIF2α PTM sites of interest have been coevolving along the eukaryotic tree. To this end, the method proposed by Pagel to detect correlated evolution on phylogenies [22] was tailored to meet the requirements imposed by the molecular model. According to this method, a likelihood ratio test was used to discriminate between two models that were fitted to the data. Both models were based on continuous-time Markov processes, one allowing only for independent evolution of the two PTM sites, the other allowing for correlated changes. The results of this analysis convincingly favored the model where both characters evolve influencing each other. To further strengthen the conclusion that these PTM sites are bona fide coevolving sites, we accounted for the uncertainty in the phylogeny by repeating the analysis on different trees obtained using diverse approaches. To this respect, we can be confident that the described coevolution of these two PTM sites is a robust conclusion with respect to slight differences in the phylogeny employed (Table 2).

The statistical evidence that these two traits (PTM sites) coevolve across a range of species suggests that common selective pressures have been acting on the traits, which may point to a functional or adaptive relationship between them. Such conclusion was further supported by two additional observations. Firstly, the co-localization of a phosphorylatable residue at position 218 and a sulfoxidable methionine at 222 has emerged several times over the evolution of eukaryotes (Figs. 5 and 6), and secondly, something more than a mere structural effect seems to be behind the observed coevolution of these sites (Table 3 and Additional file 1: Figures S2 and S3). Indeed, it is expected that residues that are close in the spatial structure of the protein will mutually influence their evolution. At such sites, a substitution that partly destabilizes the protein structure could be compensated by a subsequent change at an adjacent site to restore the stability. However, the results summarized in Table 3 suggest a functional, rather than structural, relationship between Ser-218 and Met-222. Hence, we hypothesize that the oxidation status of Met-222 allows protein kinases to monitor oxidative stress and subsequently to code this information in terms of Ser-218 phosphorylation. At this juncture, we wondered whether the presence/absence of a phosphorylatable residue at position 218 would influence the strength of the selective pressure acting on position 222, or, alternatively, whether possessing a sulfoxidable methionine at the position 222 promotes the gain/maintenance of a phospho-acceptor at 218. To examine these potential scenarios, we performed a number of analyses using reduced models. For instance, the hypothesis that the presence of a phosphorylatable residue favors the gain of methionine, was investigated by testing whether the rate of the transition parameter q_34_, (1,0) → (1,1), differed from the rate of the transition parameter q_12_, (0,0) → (0,1). Unfortunately, with the data at hand, there was not sufficient evidence to reject the null hypothesis for any of the examined reduced models (results not shown). Therefore, although the results from the current work strongly support the conclusion that both PTM sites have been coevolving, we failed to identify the probable temporal ordering of changes in these two traits.

Methionine, a relatively hydrophobic amino acid, can be found as a specificity determinant in a number of protein kinase substrate motifs [35]. Although methionine can occupy any position within these canonical recognition motifs, it is most often found at 1 or 4 positions carboxyl-terminal to the phosphorylatable serine (P + 1 and P + 4, respectively). On the other hand, oxidation of methionine at these positions has been described as a process highly selective, as opposite to random [9]. Since the oxidation of methionine to methionine sulfoxide converts the side chain of this amino acid from hydrophobic to polar and increases the capacity for H-bonding [48], the redox state at positions P + 1 and P + 4 can impact the recognition of these protein substrates by their cognate protein kinase and/or phosphatase, providing, in this way, a mechanistic coupling between oxidative signals and phosphorylation status. Interestingly, when, in a previous study, we carried out GO analysis to gain insight into the processes that may be regulated by crosstalk between Ser/Thr phosphorylation and sulfoxidation of methionine at P + 1 or P + 4, it turned out that the occurrence of MetO near phosphoserine was more prevalent in proteins related to control of translation and stress related proteins. In addition, a small set of proteins related to SGs was identified as potential target for crosstalk between sulfoxidation and phosphorylation. Therefore, in the current study we extended the coevolutionary analysis described for eIF2α to this set of stress-related proteins. For the four analyzed proteins, their respective pairs of PTM sites (phosphorylatable serine and sulfoxidable methionine at either P + 1 or P + 4) were found to be evolving in a correlated fashion (Table 4), which again suggests a relevant role for methionine sulfoxidation and serine phosphorylation crosstalk in response to oxidative stress. Overall, the findings described in this study should encourage further systematic biochemical and genetic studies aimed at understanding the role of methionine sulfoxide in the control of protein translation.

## Conclusions

Protein-bond methionine sulfoxidation was initially perceived as an inevitable damage derived from aerobic metabolism. However, this view of methionine as a vulnerable residue representing the Achilles’ heel of proteins has been gradually changing since in the 1990s Levine and coworkers proposed a role in the antioxidant defense for methionine residues as ROS scavengers More recently, the sulfoxidation of certain specific methionine residues is emerging as a posttranslational modification capable of regulating protein activity during stress conditions. In this line, we have shown that the oxidation of methionines housed within phosphorylation motifs is a highly selective process among stress-related proteins. In the current study, using evolutionary models based on continuous-time Markov chains, we have addressed the interrelationship between phosphorylation and sulfoxidation in four proteins related with the SGs. We have found their respective pairs of phosphorylatable-sulfoxidable PTM sites to be evolving in a correlated fashion through the eukaryotic lineage, which suggests a relevant role for serine/threonine phosphorylation and methionine sulfoxidation crosstalk in the control of protein synthesis during stress conditions.

## Additional file

Additional file 1: Figsures S1-S3 and Appendix A. (DOCX 8061 kb)

## Abbreviations

CTD: C-terminal domain
eIF: Eukaryotic initiation factor
GO: Gene ontology
LRT: Likelihood ratio test
MetO: Methionine sulfoxide
ML: Maximum likelihood
MP: Maximum parsimony
MSA: Multiple sequence alignment
NJ: Neighbor joining
NTD: N-terminal domain
PTM: Post-translational modification
SGs: Stress granules
