# Supplementary material for: Inferring Methionine Sulfoxidation and serine Phosphorylation crosstalk from Phylogenetic analyses
Source: BMC Evol Biol. 2017 Jul 27;17:171. doi: 10.1186/s12862-017-1017-9 (PMC5530960; doi:10.1186/s12862-017-1017-9)
Supplement: Additional file 1: — Figsures S1-S3 and Appendix A. (DOCX 8061 kb) [file 12862_2017_1017_MOESM1_ESM.docx]

**Inferring Methionine Sulfoxidation and Serine Phosphorylation Crosstalk from Phylogenetic Analyses**

Juan Carlos Aledo

Departamento de Biología Molecular y Bioquímica. Facultad de Ciencias. Universidad de Málaga. 29071-Málaga, Spain.

Figure S1A

Figure S1B

Figure S1C

Figure S1D

**Figure S2. Lack of relationship between 3D distance and LRT.** Scatter plot of 3D distance versus LRT, for all the residue pairs indicated in Table 3 from the main text.

**
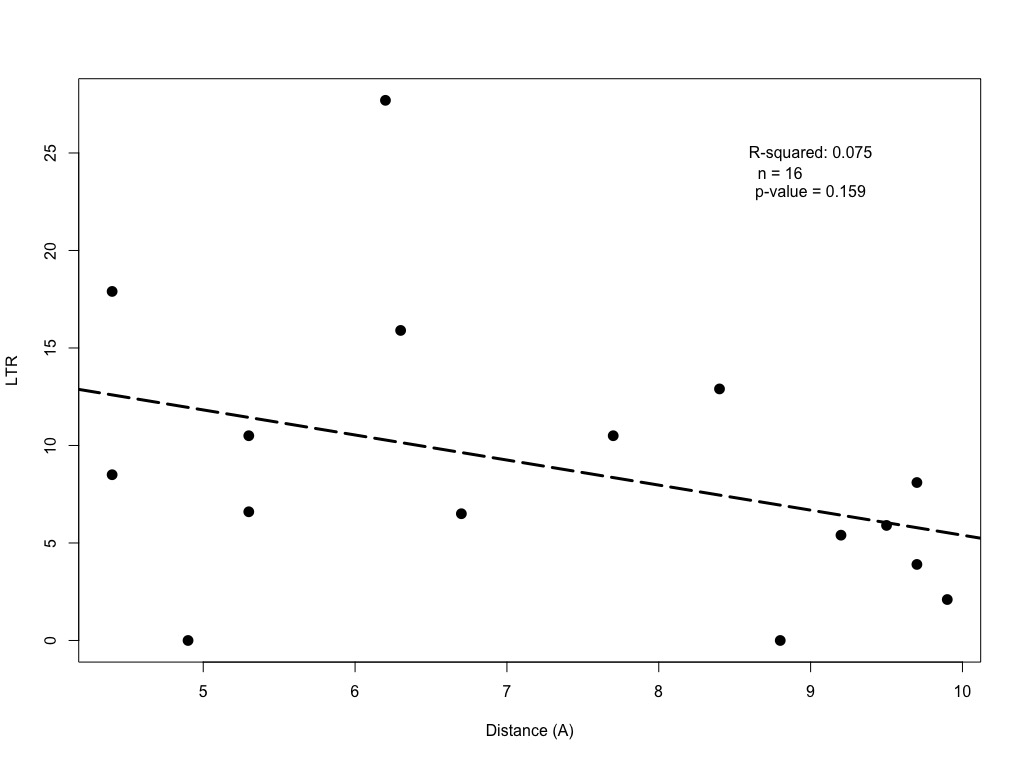
**

**Figure S3. Thermodynamic stability change versus evolutionary codependence.** Scatter plot of the mean ΔΔG versus LRT, for all the residue pairs indicated in Table 3 from the main text.

**
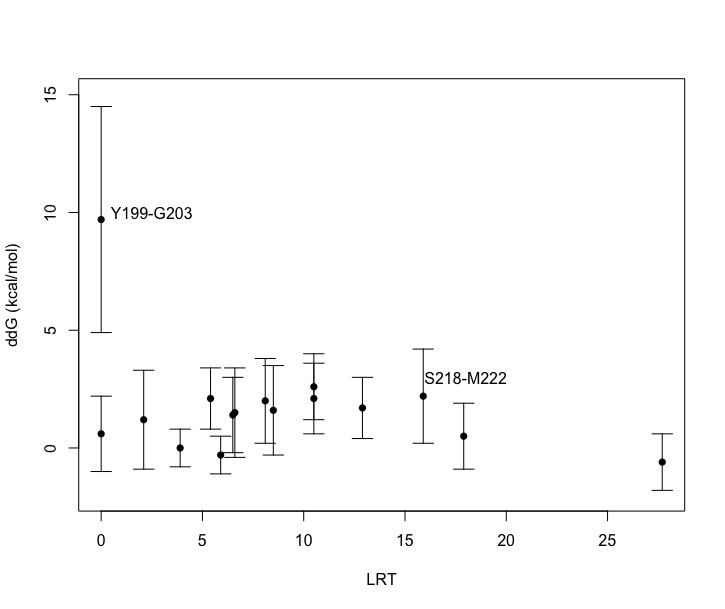
**

**Appendix A. Maximum Likelihood Estimate**

To illustrate the calculations involved, let us assume that we deal with an awfully simple data set (Fig. A1), consistent in three operational taxonomic units (OTUs), which states for the combined characters are known (3, 4, 1, for OTU-1, OTU-2 and OTU-3, respectively). In addition, as part of our data, we also know the phylogenetic relationships between these OTUs, including the tree topology and the branch distances. However, we do not know the character state at the hypothetical taxonomic unit (HTU) *a* and *b*. On the other hand, as we have seen, our markovian model is fully specified by eight rate parameters: $\Theta=\{q_{12}, q_{13},q_{21}, q_{24},q_{31}, q_{34},q_{42}, q_{43}\}$.

**Figure A1.** Hypothetical data set that will be used to illustrate how to obtain maximum likelihood estimates of the model parameters.


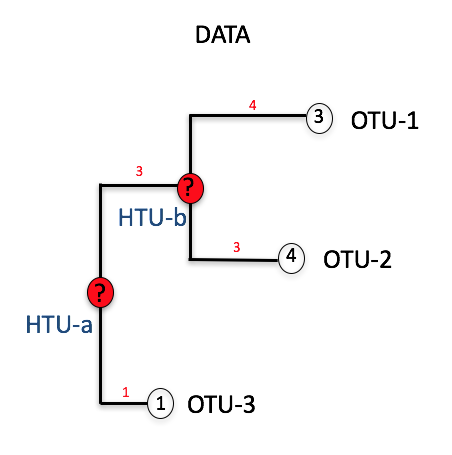


In general, the likelihood of a model is proportional to the probability of observing the data, given the values of the model parameters.

$L\left( \Theta\right)\propto P\left( DATA | \Theta\right)$ Eq. A1

Thus, we face the task of, assuming that our model is correct, finding the probability of ending up with the observed data. To this end, we will have to consider all possible assignment of character state at the internal nodes. In our purposely silly example, with only two internal nodes, all the possible realizations encompass 16 different possibilities (Fig. A2).

**Figure A2**. Considering all the possible assignment of character state at the internal nodes implies to contemplate 16 different potential realizations.


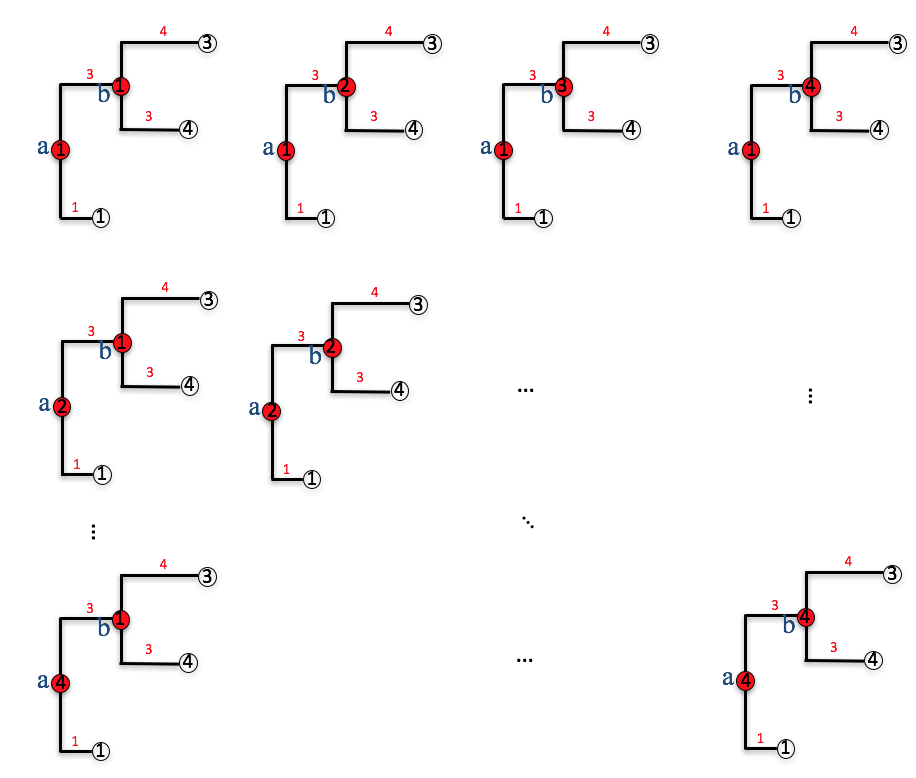


The probability of each of these single realizations is given by the product over all of the branches of the tree of the appropriate probabilities. Since in our working example we only have 4 branches, the probability of each realization will be given as a product of 4 terms. That means, that the probability of ending up with the observed data, can be written as a summation containing 16 addends, each one being the product of four terms (Fig. A3)

**Figure A3.** The likelihood can be expressed as the summation over all the possible realizations.


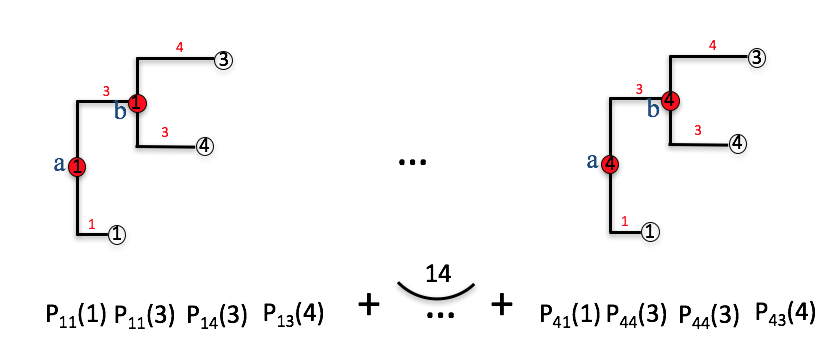


Therefore, the likelihood function that we are looking for can be written as:

$L\left( \Theta\right)= \sum_{a=1}^{4} \sum_{b=1}^{4} P_{a1}(1)P_{ab}(3)P_{b4}(3)P_{b3}(4)$ Eq. A2

Where P_ij_(t), once the time has been fixed, can be contemplated as a function of the model parameters (see Eq. A3).

$\boldsymbol{P}\left( t \right)= \left( \begin{matrix} \begin{matrix} P_{11}(t) & P_{12}(t) \\ P_{21}(t) & P_{22}(t) \end{matrix} & \begin{matrix} P_{13}(t) & P_{14}(t) \\ P_{23}(t) & P_{24}(t) \end{matrix} \\ \begin{matrix} P_{31}(t) & P_{32}(t) \\ P_{41}(t) & P_{42}(t) \end{matrix} & \begin{matrix} P_{33}(t) & P_{34}(t) \\ P_{43}(t) & P_{44}(t) \end{matrix} \end{matrix} \right)= e^{\boldsymbol{Q}t}$ Eq. A3

Therefore, the likelihood can be seen as a function of these parameters. In this way, the unknown parameters can be estimated by maximizing the likelihood function using standard numerical methods. Furthermore, the evaluation of the likelihood function for the parameter values that maximize it, measures the fit of the model to data. This procedure allows to compare two models by comparing their likelihood values (see Eq. 1 from the main text).
